# Expanding Access to Depression Treatment in Kenya Through Automated Psychological Support: Protocol for a Single-Case Experimental Design Pilot Study

**DOI:** 10.2196/11800

**Published:** 2019-04-29

**Authors:** Eric P Green, Nicholas Pearson, Sathyanath Rajasekharan, Michiel Rauws, Angela Joerin, Edith Kwobah, Christine Musyimi, Chaya Bhat, Rachel M Jones, Yihuan Lai

**Affiliations:** 1 Duke Global Health Institute Duke University Durham, NC United States; 2 Jacaranda Health San Francisco, CA United States; 3 Jacaranda Health Nairobi Kenya; 4 X2 AI Inc San Francisco, CA United States; 5 Moi Teaching and Referral Hospital Eldoret Kenya; 6 Africa Mental Health Research and Training Foundation Nairobi Kenya

**Keywords:** telemedicine, mental health, depression, artificial intelligence, Kenya, text messaging, chatbot, conversational agent

## Abstract

**Background:**

Depression during pregnancy and in the postpartum period is associated with a number of poor outcomes for women and their children. Although effective interventions exist for common mental disorders that occur during pregnancy and the postpartum period, most cases in low- and middle-income countries go untreated because of a lack of trained professionals. *Task-sharing* models such as the *Thinking Healthy Program* have shown great potential in feasibility and efficacy trials as a strategy for expanding access to treatment in low-resource settings, but there are significant barriers to scale-up. We are addressing this gap by adapting *Thinking Healthy* for automated delivery via a mobile phone. This new intervention, *Healthy Moms*, uses an existing artificial intelligence system called *Tess* (*Zuri* in Kenya) to drive conversations with users.

**Objective:**

The objective of this pilot study is to test the *Healthy Moms* perinatal depression intervention using a single-case experimental design with pregnant women and new mothers recruited from public hospitals outside of Nairobi, Kenya.

**Methods:**

We will invite patients to complete a brief, automated screening delivered via text messages to determine their eligibility. Enrolled participants will be randomized to a 1- or 2-week baseline period and then invited to begin using Zuri. Participants will be prompted to rate their mood via short message service every 3 days during the baseline and intervention periods. We will review system logs and conduct in-depth interviews with participants to study engagement with the intervention, feasibility, and acceptability. We will use visual inspection, in-depth interviews, and Bayesian estimation to generate preliminary data about the potential response to treatment.

**Results:**

Our team adapted the intervention content in April and May 2018 and completed an initial prepilot round of formative testing with 10 women from a private maternity hospital in May and June. In preparation for this pilot study, we used feedback from these users to revise the structure and content of the intervention. Recruitment for this protocol began in early 2019. Results are expected toward the end of 2019.

**Conclusions:**

The main limitation of this pilot study is that we will recruit women who live in urban and periurban centers in one part of Kenya. The results of this study may not generalize to the broader population of Kenyan women, but that is not an objective of this phase of work. Our primary objective is to gather preliminary data to know how to build and test a more robust service. We are working toward a larger study with a more diverse population.

**International Registered Report Identifier (IRRID):**

DERR1-10.2196/11800

## Introduction

### Background

Depression is a leading cause of disability worldwide. Women suffering from perinatal depression are a particularly underserved population. Depression during pregnancy and in the postpartum period (perinatal depression) affects as many as 20% of women in high-income countries [1] and maybe more in low-income countries [2]. The condition is associated with a number of poor outcomes for women and their children, including increased maternal morbidity and mortality [3,4], poor infant health [5-9], and poor developmental outcomes [10-12].

Although effective interventions exist for common mental disorders that occur during pregnancy and the postpartum period [13], most cases in low- and middle-income countries (LMICs) go untreated. In these settings, more than 7 out of 10 people who need treatment cannot access care because of a lack of trained professionals [14]. In Kenya, for example, there are only 180 psychiatric nurses outside of the capital city, a ratio of 1 provider per 200,000 people. To close this gap, the World Health Organization developed the Mental Health Gap Action Programme intervention guide outlining how to deliver mental health services in primary health care settings through nonspecialist providers. This *task-sharing* approach has proven efficacious, particularly for maternal mental health [15].

A prime example is the 15-session *Thinking Healthy Program*, a cognitive behavior therapy–based intervention for treating perinatal depression that is intentionally nonstigmatizing [16]. Community health workers—typically women educated through secondary school with no specific background in mental health—are trained over 5 to 10 days to help pregnant women learn 3 skills: (1) to identify unhealthy thinking, (2) to replace unhealthy thinking with helpful thinking, and (3) to practice thinking and acting healthy. In a trial in Pakistan with 900 pregnant women, Rahman et al found that the intervention halved the prevalence of major depression [17], and a 7-year follow-up study reported some spontaneous recovery among the control group but also a persistent effect of treatment [18].

Despite this impressive evidence of feasibility and efficacy, however, there are significant barriers to scale-up [19], and there is evidence that intervention effects might not extend to children of depressed mothers without additional engagement [20]. Common implementation challenges of task-sharing models such as *Thinking Healthy* include a lack of funding and infrastructure for training and service delivery, workforce retention in the absence of compensation or incentives for nonspecialists, high workloads, transportation costs, appointment scheduling logistics, and inadequate clinical supervision [21]. Although it is critical to study how to optimize and scale these task-sharing approaches—and a peer-delivered version of *Thinking Healthy* offers a potential cost-effective first-line strategy for treating perinatal depression [22]—the fact remains that, today, most women in LMICs who need treatment still have no access to care. It can also be argued that there are substantial service gaps in high-income countries such as the United States where it was recently recommended that all women at increased risk for perinatal depression be referred for *preventive* counseling [22].

Given this demand and barriers to scale-up, our idea is to make it possible for anyone with a basic phone to receive high-quality, evidence-based psychological support anytime, anywhere. We will do this in the context of perinatal depression by adapting *Thinking Healthy* to an existing artificial intelligence (AI) system for automated psychological support called *Tess* (which we have named *Zuri* in Kenya). This idea is innovative because it introduces an entirely new delivery channel that has the potential for a step change in expanding access to care, while also potentially augmenting and strengthening existing task-sharing models.

Zuri works by engaging a patient in conversation via a variety of trusted channels, including text messaging (short message service [SMS]). Either Zuri or the patient can start a conversation, and Zuri can be programmed to walk a patient through a structured curriculum such as *Thinking Healthy*. As a safety measure, conversations with patients in need of additional support can be handed over to live counselors as needed. Benefits of this approach include on-demand 24/7 access for an unlimited number of patients, no scheduling of appointments, no travel costs to appointments, enhanced sense of privacy and avoidance of social stigma, and high fidelity to treatment.

### Scientific Objectives and Significance

Our long-term goal is to expand access to high-quality, on-demand treatment services to people in emerging markets who suffer from common mental disorders such as perinatal depression but cannot receive care from mental health professionals because of cost and human resource constraints. The main objectives of this proposed work are to adapt *Thinking Healthy* for dissemination in Kenya through the Zuri AI system; develop and test study procedures; and generate preliminary evidence of feasibility, acceptability, and response to treatment. If successful in future full-scale trials, we will create an opportunity to expand access to evidence-based treatments on an order of magnitude that has proven difficult to achieve through traditional approaches that rely on expanding the lay and professional mental health workforce.

### Expected Outcomes

The expected outcomes of this proposed work include the following: (1) experience recruiting, screening, and enrolling women from this population; (2) evidence on feasibility and acceptability of the intervention in this setting; (3) preliminary evidence on response to treatment; and (4) a set of open source resources for automated delivery of the intervention that can be adapted for new contexts. We plan to use the preliminary evidence generated by this project to inform the design of a randomized controlled trial.

## Methods

### Research Design

We propose to adapt *Thinking Healthy* for the Zuri AI system and evaluate the combined perinatal depression intervention (which we are calling *Healthy Moms*) with a cohort of pregnant women and new mothers recruited from 2 large public hospitals in Kenya. We will use a single-case experimental design (partially nonconcurrent multiple baseline [23], open label) and qualitative interviews to generate preliminary data on feasibility, acceptability, and response to treatment.

### Participants and Recruitment

We will recruit pregnant women and new mothers from 2 large public hospitals in Kiambu County, Kenya. Both hospitals are part of a county-wide partnership offering patients innovative SMS programs that promote healthy motherhood [24]. When a woman signs up for the county SMS service, we will send her an invitation via SMS to complete an automated SMS screening (in English) to see if she is eligible for *Healthy Moms*. The screening will include questions about age, maternity status, expected or actual delivery date, 9 questions about symptoms of depression from the Patient Health Questionnaire-9 (PHQ-9) [25], and a question about her current mood.

We will inform all women who complete the screening that a study team member will call them within 1 business day. During this follow-up call, women who endorsed having thoughts of self-harm in the past 2 weeks (Question 9 on the PHQ-9) will be offered a referral for counseling but will not be eligible to enroll in *Healthy Moms* given the early stage of intervention development. All other women will be eligible to enroll as long as they confirm that they are at least 20 weeks pregnant or no more than 6 months postpartum. The study representative—a Kenyan woman fluent in English and Swahili—will assess each woman’s English-speaking ability on the call and ask women to rate their ability to read and understand English. We will allow women to enroll regardless of language ability to examine the relationship between ability and engagement, but we will inform low (English) literacy women that they might not find value in the current version of the program if they are not comfortable reading and writing in English.

If a woman chooses to continue the enrollment process, the study representative will read the informed consent form, answer her questions, and obtain verbal informed consent to enroll. Enrollees will be asked to share information about the type of phone they use, schooling, number of dependents, marital status, and employment status. There is no cap on enrollment.

### Eligibility

To be eligible to participate, women must meet the following criteria: (1) pregnant (>20 weeks) or less than 6 months postpartum; (2) receiving antenatal or postnatal health care services from a participating hospital in Kiambu County; (3) enrolled in the county SMS program; and (4) at least 18 years of age. English language proficiency is not a requirement but will be assessed for later subgroup analysis. Likewise, endorsement of depression symptoms is not a requirement, but depression severity and mood will be assessed for later subgroup analysis. Women who endorse suicidal ideation at the time of recruitment will be ineligible to enroll in the study and will be informed about potential resources for treatment.

### Randomization to Baseline Length

As each woman enrolls in the study, she will be matched to another new enrollee of similar maternity status and randomly assigned (using a random number generator) to have a 1-week or 2-week baseline period. This will ensure that every participant has a concurrent baseline period with at least one other person.

### Outcomes and Data Collection

Outcomes will include intervention use, feasibility, acceptability, depression severity, and current mood.

We will assess intervention use by reviewing Zuri system logs to document (1) completion of *Healthy Moms* sessions and (2) patient-initiated engagement with Zuri outside of scheduled sessions.

The Zuri system logs will also inform our assessment of feasibility and acceptability; low engagement will be considered a marker of potential barriers to feasibility or a lack of acceptability. We will explore these issues by inviting users to participate in up to 3 individual interviews during the evaluation period. During these interviews, we will examine barriers to access and use that may limit the feasibility of offering this intervention at scale if not addressed. We will also examine whether there are aspects of Zuri’s *personality* and style that limit acceptability and participants’ desire to engage with the intervention. To further explore feasibility issues, we will document all contact the research team has with participants outside of the Zuri AI system and log all adverse events. We are interested in determining how much assistance or encouragement users need from the team to understand and use the automated intervention.

We will assess depression severity during the enrollment screening and throughout the intervention period via SMS. An aim of the study is to determine the frequency of assessment that is useful and acceptable to participants. At a minimum, we will attempt to have at least 2 self-ratings of depression severity representing pre and posttreatment.

To measure mood, we will ask participants to rate their feelings on a 10-point scale we created and tested with users (see Table 1).

**Table 1:** Mood rating scale.

Message	Text
1	Imagine a 10-step staircase where 1 means very sad and 10 means very happy.
2	Which step best shows how you are feeling today? Very SAD 1 - 2 - 3 - 4 - 5 - 6 - 7 - 8 - 9 - 10 Very HAPPY

**Figure 1:**
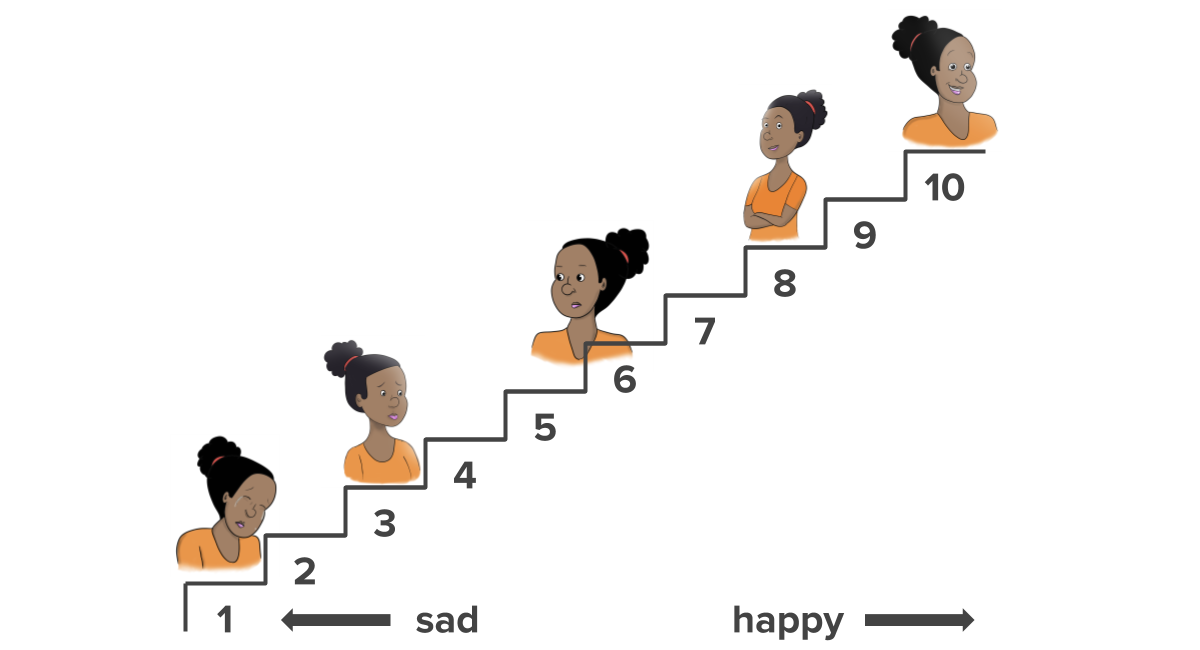
10-Step staircase visual reference for mood ratings.

We will invite women to rate their current mood via SMS during the enrollment screening and then every 3 days throughout the baseline and intervention periods. Each rating invitation will remind women of their previous rating. We will also encourage women to track and reflect on their mood and behaviors on a daily basis using the *Healthy Moms* journal we will provide as part of the intervention (not analyzed). The journal will include the 10-step illustration shown in Figure 1.

### Intervention

We will invite women to participate in up to 15 phone sessions of the *Healthy Moms* intervention depending on their maternity status at enrollment. We modeled these automated SMS sessions after the original *Thinking Healthy* manual that was developed to guide community health workers to deliver the intervention in-person over 15 sessions [16]. We also created a companion *Healthy Moms* journal that we will print and deliver to enrolled participants [26]. The journal includes modified *health calendars* from the original *Thinking Healthy* intervention along with short session summaries and writing prompts. This pilot study is an opportunity to get feedback on the journal to inform how we might adapt the content into text, audio, and video for electronic delivery (and ultimately discontinue print versions in future trials). We conducted an initial round of user testing to develop the SMS intervention journal content [27].

During each *Healthy Moms* session, women will interact with the automated system via SMS. In between sessions, women will be encouraged to start a conversation with Zuri by sending a free SMS. Zuri will attempt to discern the user’s request and respond automatically with answers or replies that employ *active listening* techniques such as restatement and reflection. If a woman discusses self-harm or other crisis topics, Zuri will alert a live study support member who can take over the chat session or call the participant directly and facilitate a referral to traditional in-person treatment if indicated. Women will be informed that the response may not be immediate at this stage of testing, so they should seek help at an emergency room if in a crisis. Participants will also be informed that they may seek concomitant care and interventions at any point during the pilot study.

Just as mental health specialists and nonspecialists trained to deliver psychotherapy improve over time with practice and experience, AI-enhanced systems such as Zuri also change, albeit in more subtle ways, given the current state of the technology. For instance, Zuri’s emotion recognition algorithms will update automatically each time it correctly or incorrectly interprets the emotional valence of a user’s input, but the didactic intervention content will not change dynamically. Modifications to the intervention content are possible but will be manual; we will review conversation transcripts and may make changes to the wording or sequence of messages if we notice that users are confused or not engaging. Any changes are expected to be minor with more substantial changes following the completion of the pilot study.

### Analysis Plan

Our analysis will seek to summarize the preliminary evidence on feasibility, acceptability, and response to treatment.

#### Describe Participant Engagement With the Intervention

**Figure 2:**
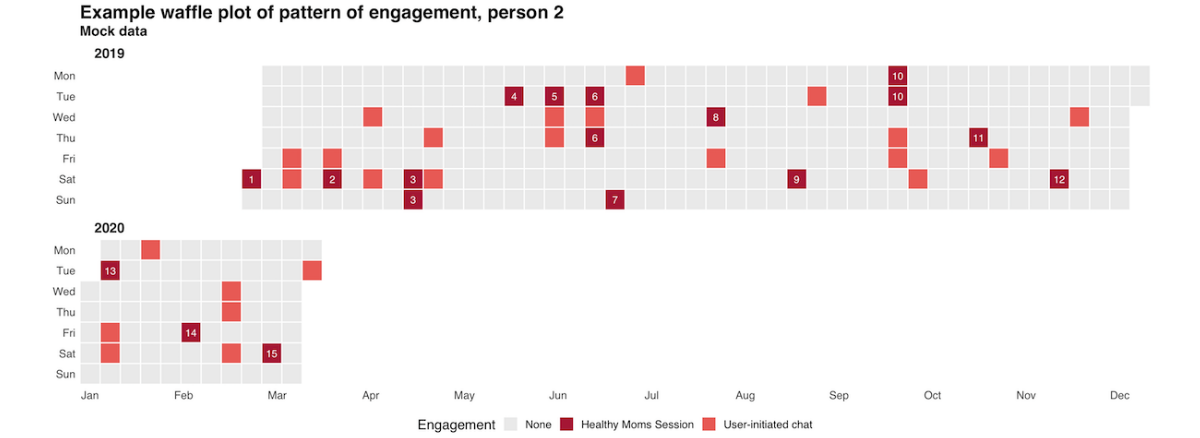
Mock waffle plot showing a hypothetical pattern of engagement for 1 participant.

We will use the system logs to summarize how frequently each participant engages with the intervention by (1) participating in a *Healthy Moms* session (in response to a scheduled invite) or (2) initiating a chat with Zuri in between scheduled sessions. Figure 2 displays a mock waffle plot that demonstrates one way we might seek to visualize these data.

As part of describing patterns of engagement, we will also calculate and summarize (1) the delay between our invitations to begin a *Healthy Moms* session and participants’ start times, (2) the proportion of *Healthy Moms* sessions started and completed, and (3) the duration of participant-initiated chats with Zuri. To further investigate the nature of participant-initiated chats, we will complete a content analysis of conversation transcripts and summarize themes.

#### Identify Potential Barriers to Intervention Feasibility and Acceptability

As a hypothesis-generating exercise, we will search for possible associations between participant characteristics measured at baseline (eg, age, education, literacy, and symptom severity) and intervention engagement. We will further explore barriers to engagement during in-depth interviews with participants and reviews of chat transcripts. Our search for barriers will include human and system factors that (1) make it challenging for participants to engage with the intervention (usability and feasibility) or (2) lower participants’ desire to engage with the intervention (acceptability).

#### Generate Evidence About the Variability in Participant Response to Treatment

**Figure 3:**
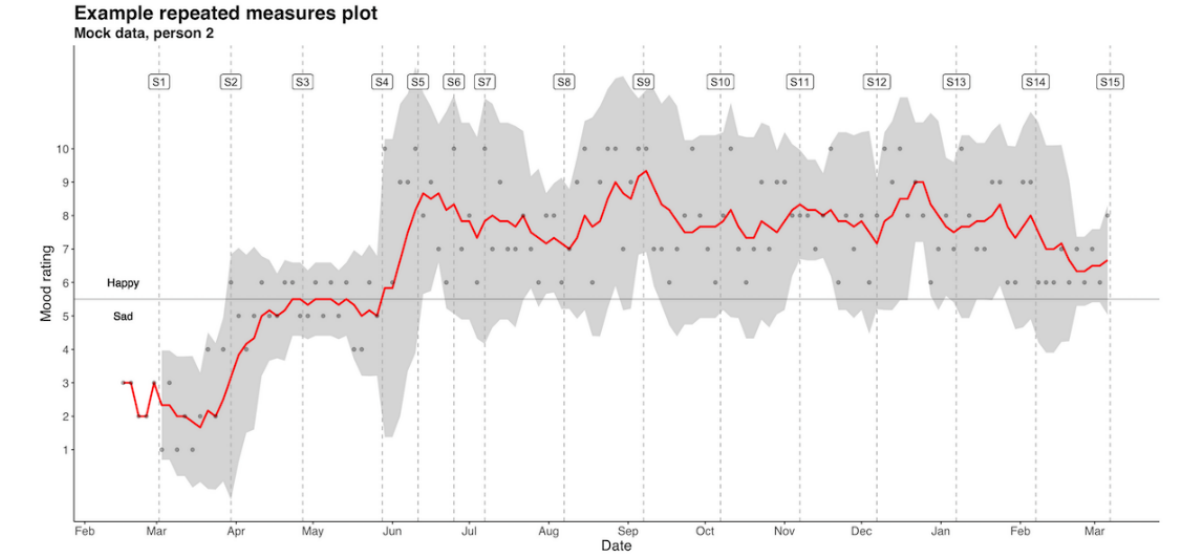
Mock plot of hypothetical mood self-ratings over time for 1 participant session completion dates. The points represent each daily mood rating, the solid red line in the postbaseline period represents a rolling mean of daily ratings (window 6), and the gray band represents the 95% CI of this rolling mean. S#: session number.

We will use visual inspection, in-depth interviews, and Bayesian estimation to generate preliminary data about the potential response to treatment. First, 2 raters will visually examine time series plots of self-ratings for within-subject changes in trends, as shown in Figure 3.

Second, during in-depth interviews with participants, we will explore potential links women see between engagement with the intervention and their mood, health, and relationships. For women who do not seem to respond, we will examine the possibility that (1) future changes to the intervention may generate a response or (2) there are clinical subtypes that may not benefit much from an intervention like *Healthy Moms*.

Third, we will attempt to aggregate the individual N-of-1 studies and estimate the magnitude of response and quantify uncertainty by fitting a Bayesian linear mixed-effects model [28-30]. The model will include a random effect for observations nested within participants and the following fixed effects: (1) an intercept, (2) a dummy indicator for the treatment phase, (3) a time-within-baseline variable centered around the first observation (equal to 0 for observations outside of the baseline period), and (4) a time-within-treatment variable centered around the last observation (equal to 0 for observations outside of the treatment period). We will apply a first-order autoregressive structure on the covariance matrix for the within-person residuals to account for autocorrelation. With these fixed effects—and with this centering—the intercept weeks will represent the mean value of the outcome at the first baseline assessment, the treatment indicator will be a contrast between the first baseline assessment and last observation in the treatment period, and the time-within-period variables will estimate linear change during the baseline and treatment periods.

### Research Ethics

We have obtained approvals to conduct this study from the Institutional Review Boards at Duke University (US, 2018-0396) and Strathmore University (Kenya, SU-IRB 0210/18) as well as from the National Commission for Science and Technology in Kenya.

A trained research assistant (female, Kenyan) will explain the study to prospective participants via telephone and administer informed consent procedures. All eligible participants must provide oral informed consent before enrollment.

Study participants will be provided with an honorarium of Ksh 1500 (roughly US $15) delivered in 3 installments via mobile money transfer (after completing sessions 1, 5, and 10) to recognize time spent completing study assessments. Women who withdraw from the study will receive a prorated honorarium.

Data will be transferred from X2AI, the creators of the AI system that we will use to deliver *Healthy Moms*, to the research team in accordance with X2AI’s data security policies [31]. The first author will store identifiable study data on Duke’s Box.com servers. At the conclusion of the study, the first author will deidentify the data for analysis using the Safe Harbor method. Quantitative data will be fully anonymized for external sharing. Participant names will never be used in study reporting.

## Results

In April and May 2018, we adapted the *Thinking Healthy* curriculum for the Kenyan context and created new content to support automated delivery via SMS text message. In May and June 2018, we conducted an initial round of user testing with 10 nondepressed women recruited from a private maternity hospital outside of Nairobi. We documented our early testing process and learnings in a series of articles on *Medium* [27,32]. From August to December 2018, we attempted to follow a face-to-face enrollment protocol, but we found it to be too slow and potentially subject to underreporting of symptoms given the stigma attached to mental health issues in this setting. Approvals for the revised protocol described here were granted in early 2019. Results are expected toward the end of 2019.

## Discussion

The main limitation of this pilot study is that we will recruit women who live in urban and periurban centers outside of Nairobi, Kenya. The results of this study may not generalize to the broader population of Kenyan women, but that is not an objective of this phase of work. Our primary objective is to gather preliminary data to know how to build and test a more robust service. We are working toward a larger study with a more diverse population.

## Abbreviations

AI: artificial intelligence
LMIC: low- and middle-income countries
PHQ-9: Patient Health Questionnaire-9
SMS: short message service
